# Alignment in implementation of evidence-based interventions: a scoping review

**DOI:** 10.1186/s13012-021-01160-w

**Published:** 2021-10-28

**Authors:** Robert Lundmark, Henna Hasson, Anne Richter, Ermine Khachatryan, Amanda Åkesson, Leif Eriksson

**Affiliations:** 1 Department of Psychology, Umeå University, SE 901 87 Umeå, Sweden; 2 Procome research group, Department of Learning, Informatics, Management and Ethics, Medical Management Centre, Karolinska Institutet, SE 171 77 Stockholm, Sweden; 3 Unit for implementation and evaluation, Center for Epidemiology and Community Medicine, Stockholm County Council, SE 171 29 Stockholm, Sweden

**Keywords:** Alignment, Implementation, EBI, Healthcare

## Abstract

**Background:**

Alignment (i.e., the process of creating fit between elements of the inner and outer context of an organization or system) in conjunction with implementation of an evidence-based intervention (EBI) has been identified as important for implementation outcomes. However, research evidence has so far not been systematically summarized. The aim of this scoping review is therefore to create an overview of how the concept of alignment has been applied in the EBI implementation literature to provide a starting point for future implementation efforts in health care.

**Methods:**

We searched for peer-reviewed English language articles in four databases (MEDLINE, Cinahl, Embase, and Web of Science) published between 2003 and 2019. Extracted data were analyzed to address the study aims. A qualitative content analysis was carried out for items with more extensive information. The review was reported according to the preferred reporting items for systematic reviews and meta-analyses extension for scoping review (PRISMA-ScR) guidelines.

**Results:**

The database searches yielded 3629 publications, of which 235 were considered potentially relevant based on the predetermined eligibility criteria, and retrieved in full text. In this review, the results of 53 studies are presented. Different definitions and conceptualizations of alignment were found, which in general could be categorized as structural, as well as social, types of alignments. Whereas the majority of studies viewed alignment as important to understand the implementation process, only a few studies actually assessed alignment. Outcomes of alignment were focused on either EBI implementation, EBI sustainment, or healthcare procedures. Different actors were identified as important for creating alignment and five overall strategies were found for achieving alignment.

**Conclusions:**

Although investigating alignment has not been the primary focus of studies focusing on EBI implementation, it has still been identified as an important factor for the implementation success. Based on the findings from this review, future research should incorporate alignment and put a stronger emphasize on testing the effectiveness of alignment related to implementation outcomes.

**Supplementary Information:**

The online version contains supplementary material available at 10.1186/s13012-021-01160-w.

Contributions to the literature

- Although alignment is frequently suggested as important for successful implementation, it has rarely been the centerpiece of studies. Our study systematically collected evidence related to alignment from implementation studies in different health care settings. This is the first initiative to summarize existing research on alignment in conjunction with implementation of EBIs.
- Results from this study highlight the research gaps related to alignment in the context of implementation of EBIs. Based on the gathered evidence, suggestions for theoretical development and future research are provided.

## Background

Over the last years, the concept of alignment has become frequently included in implementation studies as an explanation to why implementation of an evidence-based intervention (EBI) succeeded or failed [1, 2]. Alignment can be understood as the process of creating a fit between elements of the inner and outer context of an organization or system [3]. The purpose of this inter-linking process is to have goals, strategies, systems, culture, needs, leadership, etc. pulling in the same direction, and thereby optimize chances of reaching desired outcomes [1]. In the context of implementing and sustaining EBIs, alignment can also involve creating a fit between the EBI itself and elements of the inner and outer context of an organization or system [4].

The need for considering alignment seems especially important when implementing EBIs in complex and pluralistic health care organizations, which are characterized by multiple objectives and diffuse power. Due to the complexity of these organizations, implementation efforts are often extra challenging. Assuring that elements of the organizations’ inner and outer context are aligned with each other, and with the EBI, is therefore critical for a successful implementation [4, 5]. For example, an EBI may include new work practices, and for these to become realized, they need to be aligned with current practices. In turn, both old and new practices need to be aligned with organizational objectives, to increase the chances of implementation success.

However, although alignment has repeatedly been depicted as important, it has seldom been the centerpiece of implementation studies [1, 4]. Guidance on how to consider alignment during implementation of EBI is hence sparse. The lack of guidance concerns both the alignment of the EBI with elements of the inner and outer context of a health care organization, as well as the alignment of inner and outer context elements with each other in conjunction with an EBI. Additionally, authors commonly refer to different isolated aspects of alignment depending on what is being studied (e.g., alignment of an EBI with a specific practice or policy) [1], or have placed emphasis on a specific form of alignment (e.g., inter-organizational alignment) to foster the implementation of EBIs [6]. This is also evident in frameworks commonly used to guide implementation of EBIs. For example in the consolidated framework for implementation research (CFIR) [7], creating fit between the EBI and elements of the inner context is described as important; however, the process of creating fit is not described in depth.

From a conceptual perspective, the implementation literature has to a limited extent incorporated knowledge of alignment from other disciplines. Alignment is a central theme in many business research fields, such as management, organizational behavior, manufacturing, operations, marketing, information systems, human resources, and business strategy [3, 8]. Here, the main focus has been on two dimensions of alignment: structural and social [3, 8]. The structural dimension of alignment strives to enable the different components of a system to pull towards a common objective. This is done, for example, by ensuring that no conflicts exist among goals, plans, workflows, procedures, or incentives within the organizational structure. The social dimension of alignment comprises stakeholders’ shared understanding of, commitment to, and acting toward common objectives. Social alignment thus refers to the alignment of cognitive, emotional, and behavioral aspects among the different actors in the organization [8]. These two dimensions are often seen as complementary. Consequently, achieving alignment among strategies, structures, and planning systems (i.e., structural alignment) is a vital prerequisite for working effectively toward a common goal. At the same time, it is also necessary to develop a shared understanding of, and commitment to, strategies and goals (i.e., social alignment) in order to achieve those goals [3].

Although the business research literature is informative for understanding the concept of alignment and gives insights to the mechanisms and components of an alignment process, it seldom involves descriptions of an alignment process when implementing EBIs in a health care context. Hence, considering alignment during implementation of an EBI in health care organizations or systems involves addressing the complexity of this setting. It also involves moving beyond the alignment of elements of the inner and outer context of an organization or system, by also taking into account the fit of the EBI with these elements. Thus, the aim of this scoping review is therefore to create an overview of how the concept of alignment has been applied in the EBI implementation literature to provide a starting point for future implementation efforts in health care.

## Methods

A scoping review is conducted to get an overview of a broad topic and map the existing literature so that it can serve as a foundation for future research needs [9]. This scoping review was guided by the methodology suggested by Arksey and O’Malley [10] and the additional clarifications by Levac et al. [11]. The following five steps were performed: (1) identify the research question; (2) identify relevant studies; (3) study selection; (4) chart the data; and (5) collate, summarize, and report results. The PRISMA.ScR checklist [12] was used to guide reporting (Additional file 1).

### Step 1: identify the research question

Considering implementation of EBI in a healthcare context, the following research questions guided the review:

- How is alignment defined and conceptualized?
- How has alignment been assessed?
- What structural and social elements is/should be aligned?
- What are the outcomes of alignment?
- How is/can alignment (be) achieved?

### Step 2: identify relevant studies

In collaboration with the university library at Karolinska Institutet, Sweden, a search strategy based on the research questions was developed. In an iterative search process, search terms were developed by using initially identified articles that met the inclusion criteria. When reviewing the search results, we ensured that these initially identified articles were included. The search process lasted from the beginning of February until the end of March 2019. Searches were performed in four electronic databases (MEDLINE (OVID), Cinahl (Ebsco), Embase, and Web of Science (Clarivate)). As an example, the search strategy used in Web of Science is presented in Table 1. Search strategies for all databases are available in Additional file 2. In addition, references in full-text articles were scanned for potential additional articles to include.

**Table 1:** Search strategy in Web of Science

Field labels • TS/Topic = title, abstract, author keywords and Keywords Plus • NEAR/x = adjacent within x words, regardless of order • * = truncation of word for alternate endings	
#1 TS=((alignment* or aligning*))	
#2 TS=(((organisation* OR organization* OR communit* OR strateg* OR structur* OR system*) NEAR/2 (chang* or implementation* or intervention*))) OR TS=((chang* NEAR/1 management*))	
#3 #1 AND #2	
#4 #3 Refined by: PUBLICATION YEARS:( 2019 OR 2011 OR 2018 OR 2010 OR 2017 OR 2009 OR 2016 OR 2008 OR 2015 OR 2007 OR 2014 OR 2006 OR 2013 OR 2005 OR 2012) AND LANGUAGES:( ENGLISH) AND DOCUMENT TYPES: (ARTICLE OR REVIEW)	

The search strategy aimed to identify peer-reviewed full text articles in English published between 2003 and 2019. Articles that were eligible consisted of empirical research, including case studies, study protocols, methodological papers, and conceptual/debate papers published in peer-reviewed journals. Included studies reported on alignment as a facilitation strategy and/or when alignment was identified to affect implementation or change. Studies included were both descriptive studies (e.g., study protocols) as well as results from implementation of EBIs in different types of healthcare settings (e.g., primary care, hospital care, social service, and community healthcare). By purpose, our search strategy, in terms of setting, study design and type of EBI was broad. Given the lack of gathered guidance, we wanted to encompass the potential multitude of ways that the concept of alignment has been used in the literature regarding implementation of EBIs.

### Step 3: study selection

Articles from the search process were imported to Rayyan [13]. Next, all abstracts were screened separately by two reviewers (RL and research assistant 1). At weekly meetings, conflicts detected in Rayyan were discussed between the two reviewers, and if necessary, with a third reviewer (AR or HH). If disagreement remained after discussions, the article was included for full text screening. Throughout the review process, we exercised the recommended approach for retaining a high inter-reviewer reliability when reviewing topics that may include difficult judgements [14]. In this case, primarily to make sure that selected studies included a conceptual use of alignment (i.e., that describe inter-linkage of an EBI with elements of the inner and outer context of a health care organization, or elements of the inner and outer context with each other as a consequence of implementing an EBI). Use of the term alignment to describe other phenomenon’s (e.g., that results are in alignment with previous findings, or alignment between salary and performance) were excluded.

Included articles were divided among three reviewers (EK, AÅ, and research assistant 2) and two reviewers assessed each article in full text separately. When there was disagreement between the two reviewers, a third reviewer read the article and the article was discussed by all the authors at a weekly meeting as a learning opportunity and for reaching a consensus decision. Almost one-third of the articles (70 out of 235) were read by three reviewers and discussed by all authors.

### Step 4: chart the data

A data charting guide was developed by all the authors in Excel and as a first step nine articles were selected for full-text reading and tested independently by five of the authors (RL, AR, EK, AÅ, and LE). Two authors (EK and AÅ) read and extracted data from all nine articles, while the others (RL, AR, and LE) reviewed four articles each. Thus, at least three reviewers reviewed each of the nine articles. Extracted data were compared and discussed among reviewers, resulting in some modifications in the data-charting guide. The final items for data charting are presented in Table 2 and their definitions in Additional file 3.

**Table 2:** Overview of items for data charting

a) Title b) Journal c) Authors d) Year published e) Country of origin f) Study setting g) Aim of evidence-based intervention h) Description of evidence-based intervention i) Study design j) Data collection k) Definitions of alignment l) Use of theory or framework related to alignment m) Levels of alignment n) Measurement of alignment o) What is/should be aligned p) Outcome of alignment q) Strategies to create and/or sustain alignment r) Actors involved in alignment	

Out of the 235 full text articles that were read, 53 remained for data extraction and synthesis. The 53 full-text articles were divided between two authors (EK and AÅ), who independently read the articles and extracted data. To begin with and as a quality control both authors (EK and AÅ) read 12 articles together with a third person (RL or LE), followed by a discussion. Besides minor disagreements that generated adjustments in the data-charting guide, there was a generally good consensus between the reviewers.

### Step 5: collate, summarize, and report results

All data were stored and handled in Excel. A synthesis of the literature was provided by summarizing items and reporting them in text, tables, and figures. For descriptive information, such as study design, we used information provided in the article. Items with more extensive information underwent an inductive qualitative content analysis inspired by Elo and Kyngas [15]. This process implied reading each extract and assigning it a code. Thereafter, for each item, codes were grouped, based on commonality, into categories at different levels. The summary and synthesis of data was handled by four authors (RL, EK, AÅ and LE). Thereafter, all other authors were consulted at regular meetings to discuss the analysis and ensure agreement about results and synthesis.

## Results

**Fig. 1:**
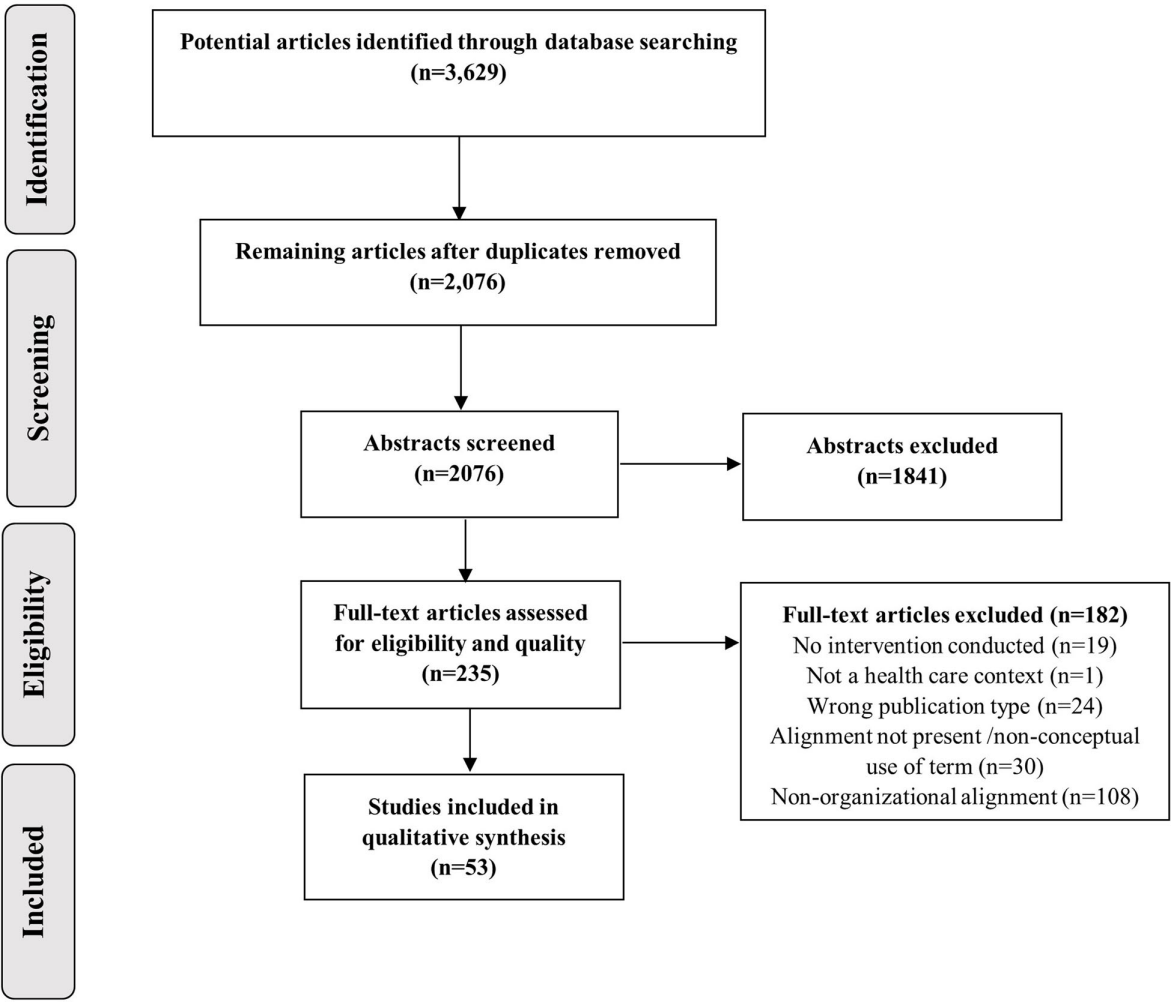
PRISMA flow diagram

The searches generated 3629 potentially relevant articles. After removal of duplicates, 2076 articles remained and underwent screening of abstracts. The screening resulted in 235 articles included for full text assessment. Finally, after the full text assessment, 53 articles were included in this review. The screening process and reasons for exclusion are presented in a PRISMA flow diagram [16] (Fig. 1).

### Study and EBI characteristics

The articles included in this review are studies with different designs (Table 3). The majority of the articles presented results for a performed EBI (*n* = 50), whereas three studies planned to evaluate an EBI (study protocols). The most common study designs were case study and cross sectional study. A majority of the studies (62.3%) used a single data collection method, where the most common method used was interview, followed by survey, document review, and observation. When multiple data collection methods were used (37.7%), the most common combinations were interview, together with survey, followed by interviews together with document review. Four studies used multiple methods with other types of combinations of data collection methods.

**Table 3:** Characteristics of the studies and EBIs presented in the articles included in the review

Study and EBI characteristics		Number of studies (Total *n* = 53)	% of total number of studies
**Study design**			
	Case study	19	35.8
	Cross-sectional	18	34.0
	Longitudinal	10	18.9
	Randomized controlled trial	4	7.5
	Other^1^	2	3.8
**Data collection methods**			
	**Single methods**	**33**	**62.3**
	Interview	24	45.3
	Survey	5	9.4
	Document review	2	3.8
	Observation	2	3.8
	**Multiple methods**	**20**	**37.7**
	Interview and survey	8	15.1
	Interview and document review	8	15.1
	Other^2^	4	7.5
**Study setting**			
	Hospital care	23	43.4
	Administrative system	14	26.4
	Primary care	6	11.3
	Community based organization	6	11.3
	Home care	4	7.5
**Type of EBI**			
	Strategies/practices	32	60.4
	Programs/model	21	39.6
**Aim of EBI**^3^			
	Improving health outcomes:		
	-In an organization	25	47.2
	-In a population	14	26.4
	Health system development	13	24.5
	Reorganization of care services	3	5.7

^1^Cross case comparison [17] and action research [18]

^2^Different combinations of methods (interview, document review, observation, survey, register)

^3^More than 100 % because some EBIs had several aims and exist in several categories

The majority of the studies were carried out in North America (*n* = 28, 52.8%), followed by Europe (*n* = 11, 20.8%), Oceania (*n* = 6, 11.3%), Africa (*n* = 7, 13.2%), and Asia (*n* = 1, 1.9%). The included studies described the implementation of various types of EBIs, mostly in a hospital care setting (Table 3). A majority of the articles focused on the implementation of strategies/practices, and within that group, about one-third (*n* = 11) were different types of e-health initiatives [17, 19–28]. Almost half of the EBIs tried to improve health outcomes in an organization, whereas the remaining EBIs targeted health outcomes on population level, health system development, and reorganization of care services. A detailed list of the study characteristics of the 53 included articles is available in Additional file 4.

### How is alignment defined and conceptualized?

All the included articles referred to alignment as an important factor to be considered during implementation of an EBI and/or as an explanation of findings (i.e., either as a lack of, or as an important part of reaching results). In most of the studies alignment referred to elements *within* an organization (*n* = 33, 62.3%), *between* organizations (*n* = 15, 28.3.6%) or on *health system* level (*n* = 5, 9.4%) (Additional file 5, column 5). However, a clear definition of alignment was seldom provided. Of the 53 included articles, 12 provided a definition of alignment [6, 19, 22, 26, 29–36]. Six of the definitions were the authors’ own (i.e., no reference where given), the other six provided a reference for their definition [19, 22, 29, 33, 35, 36]. Most of these definitions either focused on a specific aspect of alignment (e.g., service charter with goals) or used a general definition of the concept (e.g., interdependency of all human, organization, and technology elements). Furthermore, of the 12 articles providing a definition of alignment, only one clearly expressed (on a general level) that the EBI should be aligned with elements of an organization [32] (see also Additional file 6).

Beyond these 12 definitions, Hilligoss et al. [1] provided a more extensive description of alignment of EBI, in which they distinguished between, structural and social alignment. Structural alignment was the alignment of surface-level structures and processes (e.g., integrating EBI with existing routines) and adjusting existing practices to align with new routines. Social alignment was human elements, such as cognitive and sociocultural aspects of stakeholders (e.g., congruence among the perceptions of different actors).

Eleven articles used an existing implementation framework (*n* = 5) and/or organizational change theories (*n* = 2), or developing a framework (*n* = 4) to facilitate the conceptualization of alignment. The consolidated framework for implementation research (CFIR) [7] was used in two articles [37, 38], and the integrated-promoting action on research implementation in health services (i-PARIHS) [39] in another [40]. One article [21] builds on organizational theory of implementation effectiveness [41], another article [6] expanded on the exploration, preparation, implementation, sustainment (EPIS) framework [42], and yet another article [37] on the national implementation research network (NIRN) frameworks [43]. Additionally, in two articles [30, 44] organizational theories (i.e., relational development system theory and goal-setting theory) were used to explicate mechanisms for enabling alignment. In one article, a conceptual model for healthcare organizational transformation, with alignment as a central component, was developed [33]. Likewise, a change model with alignment as a key factor was developed based on implicit motivational theories to clarify how alignment facilitates implementation of EBI [1]. Two articles [28, 45] developed evaluation models that included alignment as a central component.

### How has alignment been assessed?

A total of 8 out of 53 articles assessed alignment [6, 19, 26, 30, 35, 44, 46, 47], whereas the 45 remaining articles identified alignment as an important factor when analyzing, presenting, and discussing results, but without directly assessing alignment (Additional file 5, column 2). Among the eight articles assessing alignment, five [6, 19, 26, 30, 35] had a definition of alignment (Additional file 6), while the remaining three [44, 46, 47] lacked a definition. Alignment data was collected by surveys [6, 44, 46, 47], interviews [6, 26, 30, 35], observations [19, 26], and reviews of documents [26, 35]. Three studies used multiple data collection methods [6, 26, 35], whereas the other five studies used a single method. For example, Walston and Chou [44] used a single method—employee survey—to evaluate alignment at 10 hospitals. The survey measured alignment as a function of goal commitment, goal clarity, goal acceptance, goal specificity, staff participation, available skill set, and knowledge, controlling for hospital size. Another study using a single method was Zaff et al. [6] where qualitative data was collected from several community levels and alignment across levels were assessed using cross-case analysis. Nabyonga-Orem et al. [35] used predetermined parameters for alignment, and reviewed strategic planning processes to assess impact on realizing alignment and conducted interviews at different health system levels to get views on efforts to ensure alignment. Iveroth et al. [26] was another study using multiple methods—key questions were asked to respondents about their experience and understanding of information technology, strategies used, and information technology alignment. Interviews were supplemented with observations and document reviews to add richness.

### What structural and social elements is/should be aligned?

All of the included studies included information on what should be aligned. We found three types of structural dimensions: visions and goals, system and processes (e.g., workflows, and operations), and resources and competing tasks (e.g., priorities, concurrent programs). We also found three types of social dimensions: behaviors (e.g., leadership and staff actions), thoughts and emotions (e.g., values and understandings), and interpersonal aspects (e.g., culture/climate and relationships). A high degree of alignment of these structural and social dimensions with the implementation object (or each other) were, in all cases, suggested as important for implementation outcomes. Of the 53 included articles, 25 focused only on aspects of structural alignment, and eight only on aspects of social alignment. The remaining 20 included both structural and social alignment (Table 4, see also Additional file 5, column 3). Contrary to the focus of the main part of the 12 definitions of alignment (presented above), only 8 of the 53 articles focused only on alignment between inner and outer contextual elements of an organization or system with each other [31, 36, 37, 47–51]. For example, aligning leadership across organizational levels [47], or goals and cultures across organizations [37], were in these eight articles highlighted as important facilitators during implementation of the EBI. The remaining 45 articles instead focused mainly on alignment between the EBI and social and/or structural elements of the organization or system.

**Table 4:** Dimensions of elements that should be aligned with other elements and/or with the EBI (number of articles studying the main category, category, and studied elements)

Main category	Dimension	*Studied elements	References
Structural (*n* = 45)	Vision and goals (*n* = 14)	Goals (*n* = 8) Visions (*n* = 5) Objectives (*n* = 2)	[21, 28, 29, 31, 33, 46, 50, 51] [30, 38, 52–54] [46, 49]
	System and processes (*n* = 36)	Strategy/agenda/schedule/plan (*n* = 9) Processes in general (*n* = 8) System in general (*n* = 7) Operations/mission (*n* = 6) Recommendations/guidelines (*n* = 5) Workflows (*n* = 4) Practices (*n* = 3) Services (*n* = 3) Structures (*n* = 3) Incentives (*n* = 1) Indicators (*n* = 1) Mandates (*n* = 1) Standards (*n* = 1) Work tasks (*n* = 1)	[17, 26, 32, 34, 35, 53, 55–57] [1, 33, 34, 40, 52, 55, 58, 59] [19, 23, 24, 51, 60–62] [26, 27, 33, 48, 53, 61] [18, 37, 63–65] [21, 23, 25, 58] [24, 34, 66] [36, 51, 63] [1, 25, 52] [1] [45] [37] [36] [25]
	Resources and competing tasks (*n* = 10)	Priorities/focus (*n* = 6) Resources (*n* = 4) Concurrent programs (*n* = 2) Workload (*n* = 1)	[20, 33, 38, 40, 58, 67] [20, 33, 40, 50] [21, 62] [23]
Social (*n* = 28)	Behaviors (*n* = 18)	Actions/efforts in general (*n* = 10) Leadership behaviors (*n*= 7) Staff behaviors/participation (*n* = 3) Support (*n* = 2) Activities (*n* = 1) Service deliverance (*n* = 1) Use (*n* = 1)	[6, 25, 29, 30, 33, 34, 48, 50, 57, 68] [6, 34, 36, 47, 56, 68, 69] [6, 36, 59] [6, 20] [54] [22] [22]
	Thoughts and emotions (*n* = 14)	Values (*n* = 6) Understandings (*n* = 2) Attention (*n* = 1) Attitudes (*n* = 1) Expectations (*n* = 1) Interests (*n* = 1) Needs (*n* = 1) Perceptions (*n* = 1) Readiness (*n* = 1) Satisfaction (*n* = 1) Self-determination (*n* = 1) Trust (*n* = 1) Views (*n* = 1)	[1, 21, 40, 48, 52, 70] [1, 71] [1] [6] [29] [55] [30] [44] [36] [22] [36] [1] [52]
	Interpersonal aspects (*n* = 7)	Culture/climate (*n* = 3) Relationships (*n* = 2) Social dimensions (*n* = 2) Shared community values (*n* = 1)	[6, 37, 40] [19, 52] [6, 19] [38]

*Elements as mentioned in the articles

### What are the outcomes of alignment?

In the included articles, we identified different outcomes of alignment in a chain-of-effect continuum [72]. These outcomes are summarized in three categories: EBI implementation, EBI sustainment, and healthcare performance (Table 5 and Additional file 5, column 4). Most common where descriptions of alignment as a vital facilitator in the process of implementing the EBI, or as a concluded failure of EBI implementation as a result of stakeholders not considering alignment. In turn, alignment, or lack of alignment, during implementation of an EBI also affects the sustainment of the EBI and the health care performance of the organizations. Some articles included several outcomes and are therefore mentioned in more than one category.

**Table 5:** Categories of outcomes of alignment

Category	
EBI implementation (*n* = 31)	A large proportion of the included studies described alignment as a facilitator in the process of implementing an EBI, such as a program/model or a strategy/practice (Additional file 3, column 7). Out of these articles, a majority (*n* = 21) describe alignment as vital for implementation success [18, 21, 25, 27–30, 36, 37, 40, 47, 49–53, 56, 60, 63, 69, 71]. For example, implementation was facilitated by alignment of the program with organizational goals and values [21]. Additionally, Kegeles et al. [71] concluded that when actors were aligned, implementation of an EBI became more effective. Five articles in this category describe failure of implementation due to lack of alignment [19, 22–24, 55]. For example, Sorensen et al. [23] highlights a lack of alignment between EBI and existing workflow, which resulted in a barrier to implementation. Some of the articles discusses outcome of alignment from both perspectives [1, 22, 34, 58, 71], for example, that alignment is a necessity to succeed with an EBI and that misalignment can result in fragmentation, poor quality, and soaring costs of care [1].
EBI sustainment (*n* = 5)	Five studies describe alignment as an important prerequisite for sustaining the achieved change. These studies targeted both organizations [36, 54, 66] and population [59, 62].
Healthcare performance (*n* = 18)	All articles in this category described alignment as important in change efforts initiated to improve organizational performance and/or improve health care, with the overall aim to improve capacity and/or quality of care. This category contains various strategic initiatives where alignment is part of improved care performance, for example to achieve coordination of change efforts [46] or to improve change management [65]. Data in this category consists of descriptions where alignment has contributed to improvement in healthcare [1, 20, 26, 32, 33, 46, 50, 57, 61, 64, 65, 67, 68] and/or where it is claimed that alignment was necessary in order to achieve improvements in healthcare [1, 31, 34, 35, 44, 48].

### How is/can alignment (be) achieved?

Different actors and strategies were identified as central for achieving alignment. Actors that were involved in creating and/or sustaining alignment were leaders, healthcare providers, change agents, administrative staff, community actors, policymakers, patients, and others (see Table 6 and Additional file 5, column 6 for an overview). The majority of the articles mentioned involved actors from more than one group and emphasized the importance of a collaborative process to create and/or sustain alignment. The most commonly mentioned actors were leaders and healthcare providers, highlighted in more than half of the articles in this review as having a crucial role in creating and/or sustaining alignment.

**Table 6:** Categories of involved actors of alignment

Categories	
Leaders (*n* = 29)	In the original articles actors named as change management specialist, executive leader, executive director, leader, manager, project leader, project manager, senior leader, supervisor and unit heads were included as leaders in this review. The term leader was used most frequently [1, 29, 30, 34–36, 47, 50, 52–54, 56] followed by manager [22, 29, 33, 44, 64, 67, 71]. For example, project leaders were said to have a crucial role in creating alignment [31, 32, 55] and managers were described as responsible for providing employees with motivation and enthusiasm in the alignment progress [31]. Harrison et al. [53] emphasize that participation and support from upper management in a strategic initiative is key to align organizational priorities and O’Reilly et al. [47] confirms that alignment of leadership across hierarchical levels is vital for successful implementation of a new strategic initiative.
Healthcare providers (*n* = 28)	Identified healthcare providers in this group of actors were: nurse, physician, clinician, counsellor, project team member, service provider, social worker, health promotor, healthcare staff, healthcare provider/stakeholder, health system provider, coordinator, primary caregiver and inter-professional team member. Nurse appeared as the main actors involved in creating and/or sustaining alignment [13, 18, 26–30, 33, 42, 55, 57, 58, 68] followed by physicians [18, 26–28, 30, 42, 55, 57].
Change agents (*n* = 7)	In some articles, different kinds of change agents were involved actors in the process of creating and/or sustaining alignment [18, 19, 23, 29, 37, 55, 66]. A change agent was described as an actor facilitating the change process providing knowledge and support to those involved in the change [37, 55]. A change agent also evaluated project progresses and adapted change strategies if needed [55, 66]. In one study, a change agent was described as “the champion during the intervention” facilitating sustainment [66]. All articles emphasized that a change agent’s mission was to align employees, visions and practices for a positive implementation outcome.
Administrative staff (*n* = 7)	Administrative staff was a group of actors involved in creating and/or sustaining alignment. Administrators were identified as the main actor in this category [6, 21, 37, 46, 67], followed by information technology staff [21, 22, 26]. In a study by Selick et al. [37], administrative staff was part of the implementation team and contributed with their content expertise in the process of both developing and adapting tools. It was pointed out that it was a facilitating factor to have a strong inter-professional team, and lacking some expertise, e.g., the administrative staff, created challenges to perform tasks.
Community actors (*n* = 5)	Community actors refer to health promoters who are not affiliated with a specific organization, i.e. community members [30], community-collaborative stakeholders [30], community health workers [60], provincial actors [60], school stakeholders [61], and non-governmental organizations [19, 57, 60]. Main tasks for community actors were to create and/or sustain alignment between, for example, donor countries’ development efforts with local strategies and systems [19]. Two articles highlighted non-governmental organizations as important actors in the creation and maintenance of alignment [19, 60].
Policymakers (*n* = 5)	In some studies, policymakers were identified as actors in the process of creating and/or sustaining alignment. Policymakers were identified from the following actors: political leaders [18], politicians [60], Ministry of Health [57], agencies [66], and policymakers [17]. This group of actors were vital in the creation of alignment by, for example, contributing to management of demand and access and demonstrating policy alignment [18].
Patients (*n* = 3)	Three studies [17, 49, 52] describe patients as actors involved in creating alignment. Patient activation was emphasized, i.e. the importance of aligning patient's and provider's objectives to facilitate the outcome of the EBI [49].
Other (*n* = 6)	Other types of actors that contributed to creation and/or sustainment of alignment were identified, such as donors/funders [35, 57], epidemiologists [65], doctoral students [32], potential users of secondary data [17], civil liberties groups [17], and public [52].

*Note*: Not all references are included in the text for each category, in larger categories only the most frequently mentioned actors are highlighted. However, all references are available in Additional file 5

Besides actors that drive alignment (see Table 6), five categories of strategies were identified as important to achieve alignment: design and prepare, contextualize, communicate, motivate, and evaluate (Table 7 and Additional file 5, column 7). Most commonly identified categories were design and prepare and contextualize, which both contain different types of adaption of the EBI and/or the context in order to assure alignment.

**Table 7:** Categories of strategies to achieve alignment

Categories	
Design and prepare (*n* = 15)	Designing an EBI with alignment in mind increases chances of implementation success [48, 60, 67, 68]. Especially as the complexity of implementation increases with size of organization [55], number of strategic plans [35] and with poor goal specificity [44]. The involvement of staff or employees in general was also highlighted as important during preparation, as well as during implementation and sustainment of the EBI [23, 44]. To prepare by allocating time and resources [29, 33, 46, 57], having protocols emphasizing alignment [30], training staff [44], and establish groups in the organization to ensure alignment [51], was thus considered as an important strategy.
Contextualize (*n* = 12)	The need for contextualization on different levels in healthcare systems was highlighted in a couple of the included studies. For example, to be able to successfully implement future heath care reforms there is a need to anchor suggested changes with staff in the concerned context and align changes with their values [70]. On lower system levels an overall organizational preparedness [44], integration of EBIs into existing clinical practices [25, 53, 71], facilitation of relationships between organizations [30] and consideration of organizational culture [48], may contribute to achieving alignment. Wood [46] demonstrates that to succeed with an implementation, communication of change need to be tailored to the context from which the target audience derives. Both Wood [46] and Postema et al. [28] highlighted the importance of understanding trends and variation among hospitals to allow context specific adaptation beneficial for alignment. Additionally, individuals with the right contextual experience and capacity need to be lead the implementation [34, 54, 69] and staff need to be included from all levels of the organization [67]
Communicate (*n* = 7)	To communicate was raised as an important strategy in creating and/or sustaining alignment [1, 44, 60], for example in order to achieve a shared understanding of goals and strategies [6, 20, 33, 37]. Training could also be seen as a type of communication effort, for example, for example, Nazi [25] devoted part of a training to alignment of system use with clinical workflow. Also, patient activation was highlighted as an implementation strategy that may initiate and guide patient-provider discussions with the potential of aligning the priorities of patients and providers [49]. Feedback was specifically important, allowing for the refinement of plans and goals necessary for alignment within or between organizations [25, 44, 46, 65].
Motivate (*n* = 5)	Incentives were mentioned in one study as a motivator to facilitate perceptual alignment [44], while three studies highlighted motivation in general as key [1, 68, 71]. O’Reilly et al. [47] suggested that the articulation of having a new strategy in place could be seen as motivating.
Evaluate (*n* = 5)	Evaluation of alignment throughout the implementation process was concluded beneficial for creating and/or sustaining alignment [22, 24, 26, 38, 45].

## Discussion

In this scoping review, we identified 53 articles that touched upon the concept of alignment in relation to EBI implementation in health care. The studies represented a large span of settings and different types of EBIs, which may indicate that alignment as a concept is important, independent of the context or implementation object. In all included studies, alignment between an EBI and elements of the inner or outer context of the organization or system (e.g., goals or behaviors), and/or between contextual elements with each other, was considered important for outcomes. Yet, alignment was seldom clearly defined or empirically measured. Instead, most studies retrospectively considered alignment, or lack of alignment, as an important factor that could help explain outcomes. Thus, although the results from the studies included in this review indicate that alignment is important for EBI implementation and its outcomes, there is a lack of solid data to support this. We propose that future studies in implementation science could benefit of including a hypothesis on and a direct evaluation of alignment. Using rigorous study designs and proper measurement of alignment may clarify the effects of alignment on implementation outcomes, e.g., by integration of questions asking about stakeholders’ perceptions of alignment and looking at consistency of behaviors.

Depending on what is to be aligned (e.g., EBI with elements of the inner or outer context, or elements of the inner or outer context with each other), we also encourage future research to examine the relative importance of different actors and strategies. Likewise, future studies should strive to clarify what outcomes to expect depending on form of alignment, as the studies included in this review did not provide sufficient information in this regard. Additionally, only eight of the studies focused on alignment between elements of the inner and outer context with each other—across levels, functions and/or organizational boundaries. Considering the complexity and multi-level nature of the implementation process, this suggests that there is a need for more research focusing on mechanisms and effects beyond that of only aligning the EBI with specific elements of the inner and/or outer context.

In a majority of the studies included in this review, the explicit focus was on structural alignment, focusing on alignment of an EBI with the organization’s processes, as well as vision and goals, or between these structural elements with each other (e.g., processes with goals). Social alignment (i.e., alignment of behaviors, thoughts and emotions, and culture and social aspects) was somewhat less explicitly studied. At the same time, the results also show that most studies emphasized the actions of different stakeholders (e.g., leaders, healthcare providers, change agents, and administrative staff) as being important for creating and sustaining alignment. The importance of this shared process of including relevant stakeholders could thereby be viewed as an indication that social alignment is important to achieve structural alignment, and that the two forms of alignment are complementary. In other words, structural alignment (e.g., aligning the EBI with current practices or with available resources) may be necessary for the chance to act in a new way. However, for change to occur, the EBI also must be aligned with stakeholders’ perceptions (e.g., that the EBI is in line with their values and culture) and stakeholders’ perceptions aligned with each other. Considering that structural and social aspects of alignment goes hand in hand, we suggest that future research should more clearly focus on their complementation. This complementary approach could, for example, involve developing and evaluating strategies that target the inter-linking of structural and social alignment elements (e.g., between goals and behaviors). Here, organizational climate and culture literature [73], which often discusses the inter-relatedness between structural and social aspects, may be of particular interest to refer to when addressing this question.

We identified several strategies that were considered important to achieve alignment. These strategies related to the design and preparation, contextualization, communication, motivation, and evaluation of the EBI. Similar strategies have been concluded to be important elements of an EBI implementation in general [74]. Thus, strategies to achieve alignment should, perhaps, not be understood as one more thing to do, but rather as a complement that can be integrated with already suggested important implementation strategies. For example, when communicating EBI implementation goals, one could explain how successful implementation contributes to reaching the overall goals and visions of the organization. Considering alignment may also ensure a smoother implementation, as it may clarify the need to make appropriate adaptations to the EBI, or to the structures and processes of the organization or communities, where the implementation is taking place.

Some of the studies included in this review build on an implementation framework or a change theory [1, 6, 21, 30, 33, 37, 38, 40, 44]. Among these, CFIR [7], i-PARISH [39], and EPIS [42] are commonly used within implementation science and considered important to guide EBI implementation. Alignment or fit is briefly mentioned in all these frameworks. In CFIR, to create fit is brought up in relation to the innovation and the inner context (implementation climate) elements, e.g., the importance of aligning characteristics of an innovation with norms and values among individuals in the inner context [7]. In the i-PARIHS framework, the degree of fit of the innovation with existing practice and value was highlighted [39]. Further, the facilitator, which is the active ingredient of implementation in i-PARIHS, is considered responsible to oversee alignment between the innovation and the other elements (context and recipients). In the EPIS framework, fit is mentioned in relation to the innovation and the context elements [42]. In addition, there is a component called bridging factors, which recognize the connection and relationship between the inner and the outer context, and the implementation process. Although not explicitly mentioning alignment or fit in this component, we interpret alignment as central here in achieving fit of the EBI. In our review we have identified elements that should be aligned (structural and social), actors involved in alignment (e.g., leaders, healthcare providers and change agents) and strategies to achieve alignment (e.g., design, contextualize, communicate and evaluate). Altogether, our findings suggest that alignment is complex and concern several components outlined in these implementation frameworks. Thus, one potential important next step could be to integrate alignment to encompass all the connections between elements included in this review matching the included components of the implementation frameworks. For example, when designing and preparing for implementation of an EBI stakeholder could consider and assess what structural and social elements of an organization or system that the EBI needs to be aligned with to become successful. Furthermore, stakeholders could also assess whether the current alignment of elements in the organization and/or system need to be re-aligned considering the changes introduced by the EBI.

Thus, to further integrate, and put to concrete use, the findings of this scoping review we suggest a process of three major steps to guide the understanding, creation and assessment of alignment in conjunction with implementing EBIs: .

First, attention should be paid to the alignment of the EBI with structural and social dimension of the inner and outer context of an organization and/or system. We could not find any clear definition of EBI alignment in the scoped literature. However, in line with Hilligoss et al. [1], we suggest that taking a practical perspective may be useful basis for considering alignment during implementation of EBIs. A practical perspective focus on explaining how an organization and/or system move from one state to another, viewing actions as consequences of organizational and social structures [75]. As EBI alignment involves alignment in the context of change, moving beyond the traditional present state focused organizational alignment, we propose a definition that focuses on the actual alignment of the EBI. Thus, EBI alignment primarily involves *creating a fit between an EBI and structural (e.g., visions, goals, system, processes, resources, and competing tasks) as well as social (e.g., behaviors, thoughts, emotions, and interpersonal) elements of the inner and outer context of an organization or system.*

Second, implementation of an EBI, not only requires alignment of an EBI with structural and social elements of the organization and/or system, but may also involve considering re-alignment of these elements with each other (i.e., organizational alignment) to facilitate and sustain the introduced change. Implementation of an EBI can cause a ripple effect (i.e., a series of events in a system, resulting in the evolvement of new structures of interactions and new shared meanings) [76]. Therefore, activities to re-align structural and social elements of the inner and outer context may be needed as a consequence of the implementation of an EBI.

Third, considering alignment across different organizational and/or system levels and functions can be assisted by considering both a vertical—top-down— perspective (e.g., alignment between main objectives and departments’ objectives), and horizontal—sideway—perspective (e.g., between different priorities within a department) [6]. Thus, the (re-) alignment between structural and social elements should not be viewed as a process between two isolated elements, but rather as potentially involving all affected levels and functions, structurally and socially.

## Limitations

In this review, we only considered peer-reviewed articles explicitly using the term “alignment.” Terms with similar meaning, such as “collaboration,” “coordination,” or “consistency,” were therefore excluded. This may have led to the exclusion of literature potentially contributing to the understanding of the importance of interrelatedness between different variables. However, although there is some overlap between these terms, from a conceptual perspective they do not have the same exact meaning and constitute somewhat different mechanisms for creating fit between variables. Using a wider scope would also have, from our perspective, risked making this review too extensive, less comprehensible, and most importantly less theoretically substantiated. Throughout we used a rigorous process with several reviewers involved to make decisions on exclusion to ensure that set criteria were followed in order to capture the conceptual use of alignment in implementation literature.

The included studies evaluated a wide variety of EBIs in many different settings with limited commonalities in regard to content (e.g., health promotion intervention implementation on a national level in Africa and improvement effort in American hospitals). We believe that this review reflects this contextual and substantive breadth of implementation science, and as such, contributes to the understanding of how alignment can be conceptualized across settings and type of intervention. By categorizing the literature based on the type and level of intervention (Additional file 4), we have tried to facilitate readers who wish to find relevant literature for a specific form of intervention or setting.

## Conclusions

Although seldom the centerpiece of implementation studies, alignment is proposed to play an important role for outcomes of implementation of EBIs. In this scoping review, we identify the current knowledge produced on how alignment is conceptualized in the implementation field, how it has been measured, and what elements should align with the implementation object, and/or with each other. We also examine its relation to outcomes, as well as who and what activities are involved in achieving alignment. Based on these findings, we recommend that the concept of alignment be given a more profound role in the design and evaluation of healthcare EBIs.

## Supplementary Information

**Additional file 1.** PRSIMA-ScR checklist.

**Additional file 2: Table A1–A4.** Search strategies.

**Additional file 3: Table A5.** Definitions of items for data charting.

**Additional file 4: Table A6.** Study characteristics.

**Additional file 5: Table A7.** Alignment characteristics.

**Additional file 6: Table A8.** Definitions of alignment.

## Data Availability

All data generated or analyzed during this study are included in this published article and its additional files.

## Abbreviations

CBO: Community based operations
CFIR: The consolidated framework for implementation research
i-PARIHS: The integrated-promoting action on research implementation in health services framework
EBI: Evidence-based intervention
EPIS: The exploration, preparation, implementation, sustainment framework
NIRN: The national implementation research network frameworks
RCT: Randomized control trial
PRISMA-ScR: The preferred reporting items for systematic reviews and meta-analyses extension for scoping review guidelines
