# Supplementary material for: Alignment in implementation of evidence-based interventions: a scoping review
Source: Implement Sci. 2021 Oct 28;16:93. doi: 10.1186/s13012-021-01160-w (PMC8554825; doi:10.1186/s13012-021-01160-w)
Supplement: Supplementary file 2 — Additional file 2: Table A1–A4. Search strategies. [file 13012_2021_1160_MOESM2_ESM.docx]

**Additional File 2**

*Table A1*. Search strategy in Medline

| Field labels   - exp/ = exploded MeSH term - / = non exploded MeSH term - .ti,ab,kf. = title, abstract and author keywords - adjx = adjacent within x words, regardless of order - * = truncation of word for alternate endings |
| --- |
| 1. (alignment* or aligning*).ti,ab,kf. |
| 2. Organizational Innovation/ |
| 3. Change Management/ |
| 4. ((organisation* or organization* or communit* or strateg* or structur* or system*) adj3 (chang* or implementation* or intervention*)).ti,ab,kf. |
| 5. (chang* adj2 management*).ti,ab,kf. |
| 6. or/2-5 |
| 7. 1 and 6 |
| 8. limit 7 to (english language and yr="2005 -Current") |
| 9. limit 8 to (comment or congress or consensus development conference or consensus development conference, nih or editorial or interview or letter) |
| 10. 8 NOT 9 |

*Table A2***.** Search strategy in Embase

| Field labels   - /exp = exploded Emtree term - /de = non exploded Emtree term - ti,ab = title and abstract - NEAR/x = adjacent within x words, regardless of order - * = truncation of word for alternate endings |
| --- |
| #1 alignment*:ti,ab,kw OR aligning*:ti,ab,kw |
| #2 'change management'/de |
| #3 ((organisation* OR organization* OR communit* OR strateg* OR structur* OR system*) NEAR/3 (chang* OR implementation* OR intervention*)):ti,ab,kw |
| #4 (chang* NEAR/2 management*):ti,ab,kw |
| #5 #2 OR #3 OR #4 |
| #6 #1 AND #5 AND [english]/lim AND [2005-2019]/py |
| #7 #6 AND ([article]/lim OR [article in press]/lim OR [review]/lim) |

*Table A3.* Search strategy in Web of Science

| Field labels   - TS/Topic = title, abstract, author keywords and Keywords Plus - NEAR/x = adjacent within x words, regardless of order - * = truncation of word for alternate endings |
| --- |
| #1 TS=((alignment* or aligning*)) |
| #2 TS=(((organisation* OR organization* OR communit* OR strateg* OR structur* OR system*) NEAR/2 (chang* or implementation* or intervention*))) OR TS=((chang* NEAR/1 management*)) |
| #3 #1 AND #2 |
| #4 #3 Refined by: PUBLICATION YEARS:( 2019 OR 2011 OR 2018 OR 2010 OR 2017 OR 2009 OR 2016 OR 2008 OR 2015 OR 2007 OR 2014 OR 2006 OR 2013 OR 2005 OR 2012) AND LANGUAGES:( ENGLISH) AND DOCUMENT TYPES: (ARTICLE OR REVIEW) |

*Table A4.* Search strategy in Cinahl

| Field labels   - MH+ = exploded Cinahl Heading - MH = non exploded Cinahl Heading - TI = title - AB = abstract - Nx = adjacent within x words, regardless of order - * = truncation of word for alternate endings |
| --- |
| S1 TI ( (alignment* or aligning*) ) OR AB ( (alignment* or aligning*) ) |
| S2 (MH "Organizational Change") |
| S3 (MH "Change Management") |
| S4 TI ( (organisation* or organization* or communit* or strateg* or structur* or system*) N2 (chang* or implementation* or intervention*) ) OR AB ( (organisation* or organization* or communit* or strateg* or structur* or system*) N2 (chang* or implementation* or intervention*) ) |
| S5 TI (chang* N1 management*) OR AB (chang* N1 management*) |
| S6 S2 OR S3 OR S4 OR S5 |
| S7 S1 AND S6 Limiters - Peer Reviewed; Published Date: 20050101-20181231; English |
