# Supplementary material for: Alignment in implementation of evidence-based interventions: a scoping review
Source: Implement Sci. 2021 Oct 28;16:93. doi: 10.1186/s13012-021-01160-w (PMC8554825; doi:10.1186/s13012-021-01160-w)
Supplement: Supplementary file 3 — Additional file 3: Table A5. Definitions of items for data charting. [file 13012_2021_1160_MOESM3_ESM.docx]

**Additional File 3**

*Table A5.* Definitions of items for data charting

| **Items for data charting** | **Definition** |
| --- | --- |
| 1. Title | Articles full title |
| 1. Journal | Name of Journal |
| 1. Authors | Names of authors of article |
| 1. Year published | Year when article was accepted for publication |
| 1. Country of origin | Country where evidence-based intervention was conducted |
| 1. Study setting | Study setting, e.g. Primary Care, Home Care, Hospital Care and Community Based Organization |
| 1. Aim of evidence-based intervention | What is the aim of the evidence-based intervention? |
| 1. Description of evidence-based intervention | What type of evidence-based intervention is implemented? |
| 1. Study designs | Type of study design, e.g. Longitudinal study, Cross-sectional study, Case study and Randomized Controlled Trial |
| 1. Data collection | Type of data collection methods, e.g. Interview, Survey and Observation |
| 1. Definitions of alignment | Description of definition of alignment presented in article |
| 1. Use of theory or framework related to alignment | Description of theories related to alignment that have been used |
| 1. Outcome of alignment | What outcomes of alignment are described |
| 1. What is/should be aligned | Description of what is or should be aligned |
| 1. Levels of alignment | Distinction of where alignment have been performed (within organization, between organizations or in a health care system) |
| 1. Strategies to create and/or sustain alignment | Description of strategies important to create and/or sustain alignment |
| 1. Actors involved in alignment | Who is involved in alignment? |
| 1. Measurement of alignment | Description how alignment is measured |
