# Supplementary material for: Alignment in implementation of evidence-based interventions: a scoping review
Source: Implement Sci. 2021 Oct 28;16:93. doi: 10.1186/s13012-021-01160-w (PMC8554825; doi:10.1186/s13012-021-01160-w)
Supplement: Supplementary file 4 — Additional file 4: Table A6. Study characteristics. [file 13012_2021_1160_MOESM4_ESM.docx]

**Additional file 4**

*Table A6.* Study characteristics.

| **Author (year)** | **Country** | **Setting** | **Design** | **Data collection** | **Aim of EBI** | **Type of EBI** |
| --- | --- | --- | --- | --- | --- | --- |
| Abejirinde (2018) [32] | Rwanda | CBO | Case study | Interview; Document review | Improving population health outcomes | Program/Model |
| Adsul (2017) [50] | U.S | Hospital care | Cross-sectional | Interview | Improving organization health outcomes | Program/Model |
| Bayly (2018) [40] | Canada | Home care | Longitudinal | Interview | Improving organization health outcomes | Strategy/Practice |
| Buzza (2010) [49] | U.S | Primary care | Cross-sectional | Interview | Improving organization health outcomes | Strategy/Practice |
| Carroll (2015) [36] | Australia | Hospital care | Case study | Interview | Reorganization of care service | Program/Model |
| de Savigny (2012) [57] | Ghana, Tanzania | Administrative system | Case study | Interview; Document review | Improving population health outcomes | Strategy/Practice |
| Egeland (2019)* [56] | Norway | Hospital care | RCT | Survey; Interview | Health system development | Strategy/Practice |
| Fleiszer (2016) [54] | Canada | Hospital care | Case study | Interview; Document review | Improving organization health outcomes | Program/Model |
| Freeman (2018) [70] | Australia | Primary care | Case study | Survey; Interview | Reorganization of care service | Strategy/Practice |
| Gebre-Mariam (2018) [19] | Ethiopia | Administrative system | Cross-sectional | Observation | Health system development | Strategy/Practice |
| Glisson (2016) [67] | U.S | CBO | RCT | Survey | Health system development | Strategy/Practice |
| Greenhalgh (2013) [17] | U.K | Administrative system | Other ( Cross case comparison) | Document review | Improving organization health outcomes | Strategy/Practice |
| Harrison (2016) [53] | U.S | Primary care | Cross-sectional | Interview | Health system development | Strategy/Practice |
| Healey (2019) [62] | Uganda, Zambia | Primary care | Cross-sectional | Interview | Improving organization health outcomes | Program/Model |
| Hilligoss (2015) [1] | U.S | Hospital care | Case study | Interview | Improving organization health outcomes | Programs/Model |
| Iveroth (2013) [26] | Sweden | Hospital care | Longitudinal | Interview; Document review | Improving organization health outcomes | Strategy/Practice |
| Kawonga (2012) [59] | South Africa | Administrative system | Cross-sectional | Interview; Document review | Improving population health outcomes | Strategy/Practice |
| Kegeles (2015) [71] | U.S | Primary care | Longitudinal | Interview | Improving population health outcomes | Program/Model |
| Kertesz (2014) [34] | U.S | Hospital care | Case study | Interview | Improving population health outcomes | Program/Model |
| Laws (2016) [63] | Australia | Administrative system | RCT | Interview | Improving population health outcomes | Program/Model |
| Lukas (2007) [33] | U.S | Hospital care | Longitudinal | Interview; Document review | Improving organization health outcomes | Program/Model |
| Lyon (2018)* [6] | U.S | CBO | Cross-sectional | Survey; Interview | Improving population health outcomes | Program/Model |
| Margolis (2010) [68] | U.S | Administrative system | Cross-sectional | Registers; Document review | Improving organization health outcomes & Health system development | Program/Model |
| McIntyre (2019) [61] | U.S | CBO | Longitudinal | Survey | Improving population health outcomes | Strategy/Practice |
| Nabyonga-Orem (2018) [35] | Swaziland, Zanzibar | Administrative system | Case study | Interview; Document review | Health system development | Program/Model |
| Nazi (2013) [25] | U.S | Administrative system | Case study | Interview | Improving organization health outcomes | Strategy/Practice |
| Nelson (2014) [48] | U.S | Hospital care | Case study | Observation | Improving organization health outcomes | Strategy/Practice |
| Nicks (2016) [27] | U.S | Home care | Cross-sectional | Interview | Improving population health outcomes | Strategy/Practice |
| O’Reilly (2010) [47] | U.S | Hospital care | Cross-sectional | Survey | Improving organization health outcomes | Strategy/Practice |
| Piscotty (2011) [24] | Australia | Hospital care | Cross-sectional | Interview | Improving organization health outcomes | Strategy/Practice |
| Postema (2012) [28] | Netherlands | Home care | Case study | Interview | Improving organization health outcomes | Strategy/Practice |
| Pucher (2015) [65] | Netherlands | Administrative system | Longitudinal | Survey; Interview | Improving population health outcomes | Program/Model |
| Rahm (2015) [58] | U.S | Primary care | Cross-sectional | Interview | Improving population health outcomes | Program/Model |
| Reedy (2005) [45] | U.S | Administrative system | Case study | Document review | Improving organization health outcomes & Health system development | Strategy/Practice |
| Reszel (2019) [20] | Canada | Hospital care | Case study | Interview; Document review | Improving organization health outcomes | Strategy/Practice |
| Rycroft-Malone (2016) [52] | U.K | Administrative system | Longitudinal | Interview | Health system development | Program/Model |
| Sarkies (2018)* [64] | Australia, New Zeeland | Administrative system | RCT | Survey; Interview | Health system development | Strategy/Practice |
| Schneider (2014) [60] | South Africa | Administrative system | Case study | Interview | Health system development | Strategy/Practice |
| Schmit (2011) [29] | Belgium | Hospital care | Case study | Interview | Improving organization health outcomes | Strategy/Practice |
| Selick (2018) [37] | Canada | Hospital care | Cross-sectional | Interview | Improving organization health outcomes | Strategy/Practice |
| Shaw (2013) [21] | U.S | Hospital care | Cross-sectional | Survey; Interview | Improving organization health outcomes | Strategy/Practice |
| Sorensen (2011) [23] | U.S | Hospital care | Case study | Interview | Improving population health outcomes | Strategy/Practice |
| Stumbo (2017) [66] | U.S | Hospital care | Cross-sectional | Survey; Interview | Health system development | Strategy/Practice |
| Teeters (2018) [38] | U.S | CBO | Cross-sectional | Survey; Interview | Improving population health outcomes | Program/Model |
| Thomassen (2014) [31] | Netherlands | Hospital care | Case study | Interview | Improving organization health outcomes | Strategy/Practice |
| Turner (2016) [69] | U.K | Hospital care | Cross-sectional | Interview | Reorganization of care service | Program/Model |
| Vos (2010) [55] | Germany | Hospital care | Case study | Survey; Interview; Document review; Observation | Improving organization health outcomes | Strategy/Practice |
| Wade (2016) [18] | Australia | Home care | Other (Action research) | Interview | Improving population health outcomes | Program/Model |
| Walston (2006) [44] | U.S | Hospital care | Cross-sectional | Survey | Improving organization health outcomes | Strategy/Practice |
| Wood (2018) [46] | U.S | Administrative system | Longitudinal | Survey | Health system development | Strategy/Practice |
| Wright (2006) [51] | U.K | Hospital care | Longitudinal | Survey; Observation | Improving organization health outcomes | Program/Model |
| Yusof (2015) [22] | Malaysia | Hospital care | Case study | Interview; Document review; Observation | Improving organization health outcomes | Strategy/Practice |
| Zaff (2015) [30] | U.S | CBO | Longitudinal | Interview | Health system development | Program/Model |

*Note*. * Study protocol, EBI = Evidence-Based Intervention, CBO = Community Based Operations, RCT = Randomized Control Trial
