# Supplementary material for: Alignment in implementation of evidence-based interventions: a scoping review
Source: Implement Sci. 2021 Oct 28;16:93. doi: 10.1186/s13012-021-01160-w (PMC8554825; doi:10.1186/s13012-021-01160-w)
Supplement: Supplementary file 5 — Additional file 5: Table A7. Alignment characteristics. [file 13012_2021_1160_MOESM5_ESM.docx]

**Additional file 5**

*Table A7.* Alignment characteristics.

| **Author (year)** | **Assessment** | **What is/should be aligned** | **Outcomes** | **Level** | **Involved actors** | **Strategies** |
| --- | --- | --- | --- | --- | --- | --- |
| Abejirinde (2018) [32] | - | System and processes | Healthcare practice | Between | Leaders; Healthcare providers; Others | - |
| Adsul (2017) [50] | - | Vision and goals; Resources and competing tasks; Behaviors | Healthcare practice | Within | Leaders; Healthcare providers | - |
| Bayly (2018) [40] | - | System and processes; Resources and competing tasks; Thoughts and emotions; Culture and social aspects | Implementation | Within | - | - |
| Buzza (2010) [49] | - | Vision and goals | Implementation | Within | Healthcare providers; Patients | Drive alignment |
| Carroll (2015) [36] | - | System and processes; Behaviors; Thoughts and emotions | Implementation | Between | Leaders | Drive alignment |
| de Savigny (2012) [57] | - | System and processes; Behaviors | Healthcare practice | Between | Community actors; Policymakers, Others | Design and prepare; Contextualize |
| Egeland (2019)* [56] | - | System and processes; Behaviors | Implementation | Within | Leaders | - |
| Fleiszer (2016) [54] | - | Vision and goals; System and processes; Behaviors | Sustainment | Within | Leaders | Drive alignment |
| Freeman (2018) [70] | - | Thoughts and emotions | - | Within | - | Design and prepare |
| Gebre-Mariam (2018) [19] | Observation | System and processes; Culture and social aspects | Implementation | Between | Healthcare providers; Community actors | Drive alignment |
| Glisson (2016) [67] | - | Resources and competing tasks | Healthcare practice | Within | Leaders; Healthcare providers; Administrative staff | Design and prepare; Drive alignment |
| Greenhalgh (2013) [17] | - | System and processes | Implementation | Between | Healthcare providers; Policymakers; Patients; Others | - |
| Harrison (2016) [53] | - | Vision and goals; System and processes | Implementation | Within | Leaders | Contextualize; Drive alignment |
| Healey (2019) [62] | - | System and processes; Resources and competing tasks | Sustainment | Between | - | - |
| Hilligoss (2015) [1] | - | System and processes; Thoughts and emotions; Culture and social aspects | Implementation  Healthcare practice | Within | Healthcare providers | Drive alignment; Communicate; Motivate |
| Iveroth (2013) [26] | Observation  Interview  Document review | System and processes | Healthcare practice | Within | Administrative staff | Evaluate |
| Kawonga (2012) [59] | - | System and processes; Behaviors | Sustainment | System | Healthcare providers | - |
| Kegeles (2015) [71] | - | Thoughts and emotions | Implementation | Within | Leaders; Healthcare providers | Contextualize; Motivate |
| Kertesz (2014) [34] | - | System and processes; Behaviors | Implementation  Healthcare practice | Within | Leaders | Drive alignment |
| Laws (2016) [63] | - | Vision and goals; System and processes | Implementation | System | Leaders | - |
| Lukas (2007) [33] | - | Vision and goals; System and processes; Resources and competing tasks; Behaviors | Healthcare practice | Within | Leaders | Design and prepare; Drive alignment; Communicate; Evaluate |
| Lyon (2018)* [6] | Survey | Behaviors; Thoughts and emotions; Culture and social aspects | - | Between | Leaders; Healthcare providers; Administrative staff | - |
| Margolis (2010) [68] | - | Behaviors | Healthcare practice | System | Healthcare providers | Design and prepare; Drive alignment |
| McIntyre (2019) [61] | - | System and processes | Healthcare practice | Within | Community actors | - |
| Nabyonga-Orem (2018) [35] | Interview  Document review | System and processes | Healthcare practice | Between | Leaders; Others | Design and prepare |
| Nazi (2013) [25] | - | System and processes; System and processes; Behaviors | Implementation | Within | Healthcare providers | Contextualize; Drive alignment; Communicate |
| Nelson (2014) [48] | - | System and processes; Behaviors; Thoughts and emotions | Healthcare practice | Within | Leaders; Healthcare providers | Design and prepare; Contextualize |
| Nicks (2016) [27] | - | System and processes | Implementation | Within | - | - |
| O’Reilly (2010) [47] | Survey | Behaviors | Implementation | Within | Leaders | Drive alignment; Motivate |
| Piscotty (2011) [24] | - | System and processes | Implementation | Within | Healthcare providers | Evaluate |
| Postema (2012) [28] | - | Vision and goals | Implementation | Within | - | Contextualize |
| Pucher (2015) [65] | - | System and processes | Healthcare practice | Between | Leaders; Healthcare providers; Others | Communicate |
| Rahm (2015) [58] | - | System and processes; Resources and competing tasks | Implementation | Within | Healthcare providers | - |
| Reedy (2005) [45] | - | System and processes | Non declared | System | Healthcare providers | Design and prepare |
| Reszel (2019) [20] | - | Resources and competing tasks; Behaviors | Healthcare practice | Within | Healthcare providers | Drive alignment |
| Rycroft-Malone (2016) [52] | - | Vision and goals; System and processes; Thoughts and emotions; Culture and social aspects | Implementation | Within | Leaders; Patients; Others | Drive alignment; Evaluate |
| Sarkies (2018)* [64] | - | System and processes | Healthcare practice | Between | Leaders; Healthcare providers | - |
| Schneider (2014) [60] | - | System and processes | Implementation | System | Community actors; Policymakers | Design and prepare |
| Schmit (2011) [29] | - | Vision and goals; Behaviors; Thoughts and emotions | Implementation | Within | Leaders; Healthcare providers | Design and prepare; Drive alignment |
| Selick (2018) [37] | - | System and processes; Culture and social aspects | Implementation | Between | Healthcare providers; Administrative staff; Change agents | Communicate |
| Shaw (2013) [21] | - | Vision and goals; System and processes; Resources and competing tasks; Thoughts and emotions | Implementation | Within | Healthcare providers; Administrative staff | - |
| Sorensen (2011) [23] | - | System and processes; Resources and competing tasks | Implementation | Within | Healthcare providers | Drive alignment |
| Stumbo (2017) [66] | - | System and processes | Implementation  Sustainment | Within | Leaders; Change agents; Policymakers | - |
| Teeters (2018) [38] | - | System and processes; Resources and competing tasks; Culture and social aspects | Implementation | Within | - | Design and prepare; Drive alignment |
| Thomassen (2014) [31] | - | Vision and goals | Healthcare practice | Between | Leaders | - |
| Turner (2016) [69] | - | Behaviors | Implementation | Between | Leaders | Drive alignment |
| Vos (2010) [55] | - | System and processes; Thoughts and emotions | Implementation | Within | Leaders; Healthcare providers; Change agents | Design and prepare |
| Wade (2016) [18] | - | System and processes | Implementation | Within | Policymakers | Drive alignment |
| Walston (2006) [44] | Survey | Thoughts and emotions | Healthcare practice | Within | Leaders; Healthcare providers | Design and prepare; Contextualize; Drive alignment; Communicate; Motivate |
| Wood (2018) [46] | Survey | Vision and goals | Healthcare practice | Within | Leaders; Healthcare providers; Administrative staff | Contextualize; Communicate |
| Wright (2006) [51] | - | Vision and goals; System and processes | Implementation | Between | Leaders | Design and prepare |
| Yusof (2015) [22] | - | Behaviors; Thoughts and emotions | Implementation | Within | Leaders; Healthcare providers; Administrative staff | Evaluate |
| Zaff (2015) [30] | Interview | Vison and goals; Behaviors; Thoughts and emotions | Implementation | Between | Leaders; Healthcare providers; Community actors | Design and prepare; Contextualize; Drive alignment |

*Note*. * Study protocol
