# Supplementary material for: Alignment in implementation of evidence-based interventions: a scoping review
Source: Implement Sci. 2021 Oct 28;16:93. doi: 10.1186/s13012-021-01160-w (PMC8554825; doi:10.1186/s13012-021-01160-w)
Supplement: Supplementary file 6 — Additional file 6: Table A8. Definitions of alignment. [file 13012_2021_1160_MOESM6_ESM.docx]

**Additional File 6**

*Table A8.* Definitions of alignment

| **Reference** | **Definition** |
| --- | --- |
| Lyon et al. [6] ^1^ | Inter-organizational alignment (i.e., similarity in values, characteristics, activities related to implementation across organizations). |
| Schmit et al. [29] ^2^ | The congruence between the perceptions of different actors around major organizational objectives. …alignment is the result of a negotiation process aimed at reaching a consensus between actors groups in a change context. The alignment is a core concept for change management within complex organizations that have features (multiple objectives and diffuse power) that are the source of potential tensions. |
| Gebre-Mariam [17] ^2^ | In IS [information system]^3^ management, alignment is commonly defined as the fit between business strategy, IT strategy, business infrastructure, and IT infrastructure. Generally, strategic alignment is the fit between business needs and IS priorities. |
| Yusof [22] ^2^ | The essential interdependency of all human, organisation, and technology factors. |
| Iveroth et al. [26] ^1^ | Information technology (IT) alignment - the fit between an  organization’s IT and its business strategy |
| Zaff et al. [30] ^1^ | Sharing the same or complementary perceived community needs and how these needs will be met, across multiple levels of the community (e.g., community leaders, business leaders, parents, and youth). |
| Thomassen et al. [31] ^1^ | Strategic alignment: The service charter fits the vision of all involved organizations and the goals of the chain |
| Abejirinde et al. [32] ^1,4^ | Integrating [the EBI]^3^ with existing structures |
| Lukas et al. [33] ^2^ | Consistency of plans, processes, information, resource decisions, actions, results, and analysis to support key organization-wide goals…moving work at all levels of the organization in a consistent direction. |
| Kertesz et al. [34] ^1^ | Alignment is the consistency of plans, processes, information, resource decisions, actions, results and analysis to support key organization-wide goals—moving work at all levels of the organization in a consistent direction of shared purpose. |
| Nabyonga-Orem et al. [35] ^2^ | A state where actors rally around a common mission and vision to achieve targeted objectives. |
| Carroll et al. [36] ^2^ | …organisational sharing of the same values |

^1^ Authors own definition of alignment

^2^ Authors provide reference for definition of alignment

^3^ Our addition for clarification

^4^ Using a definition with focus on alignment of the EBI
